# Including anisotropy in homogenised inverse remodelling algorithms for habitual load case estimation in bone

**DOI:** 10.1007/s10237-026-02106-1

**Published:** 2026-07-09

**Authors:** Gabriela Gerber, Philippe Zysset

**Affiliations:** ARTORG Center for Biomedical Engineering Research, University of Bern, Freiburgstrasse 3, 3010 Bern, Bern Switzerland

**Keywords:** Load estimation, Inverse remodelling, Homogenised finite element, Anisotropy, Bone

## Abstract

Bone architecture, including local bone volume fraction and fabric anisotropy, is optimised to resist forces and moments induced by long-term average loading patterns. Finite element (FE)-based inverse bone remodelling allows to estimate a habitual load case using high-resolution computed tomography images. Homogenised FE models use less computational resources than micro-FE approaches but have so far been limited to density-based remodelling. This study aims to include fabric anisotropy in homogenised inverse bone remodelling algorithms for the estimation of long-term habitual loading conditions in bone. The newly developed theoretical framework was applied to 24 human distal tibia samples, and the influence of fabric anisotropy, St. Venant effect, and load case complexity on predicted forces, moments and objective function outcomes was analysed. Physiologically plausible forces and moments were obtained for all analysis types. Although the distal tibia is predominantly loaded in axial compression, the inclusion of additional forces and moments improved the optimisation quality by reducing the overall objective function (*p* < 0.001). In contrast, global optimisation outcomes were not significantly affected by the exclusion of boundary layer elements from the optimisation scheme despite the occurrence of relatively high stress discrepancies in these regions. Including microstructural orientation into the inverse bone remodelling algorithm led to a significant increase in the predicted load magnitude (*p*  < 0.001) and a reduction in objective function values by approximately 50 %, suggesting improved agreement between the predicted load case and bone architecture. These findings underline the relevance of considering both bone volume fraction and fabric anisotropy for habitual load case estimation in bone using homogenized FE.

## Introduction

Bone morphology is closely tied to its mechanical function. The skeletal system provides structural support to the body, serves as an attachment point for muscles to enable locomotion, and protects the inner organs (Clarke 2008). In this context, bones must be strong enough to sustain the forces and moments experienced under physiological loading conditions without the formation of excessive bone mass in order to preserve vital energy and nutrients (Currey 2012). The light-weight design of bone with its cortical shell and trabecular structures following the directions of principal stress within the bone allows it to meet these requirements, while at the same time, bone mass is minimised (Wolff 1893; Fyhrie and Carter 1986). Bone architecture is continuously adapting to the mechanical environment, with bone matrix being formed in areas of high tissue loading, and bone resorption taking place in both areas with low mechanical stimulation as well as in excessively loaded regions (Frost 1987; Cardoso et al. 2009). This so-called mechanostat theory is supported by empirical evidence from multiple studies, where changes in bone mass were observed following limb immobilisation (Dauty et al. 2000; Friedman et al. 2019), prolonged bedrest (Beller et al. 2011; Rittweger et al. 2005), exposure to microgravity (Fu et al. 2021; Stavnichuk et al. 2020) or increased levels of physical activity (Guadalupe-Grau et al. 2009). However, contrary to the original mechanostat theory (Frost 1987), the existence of a stimulus window which does not trigger bone formation or resorption was not confirmed by experimental results in humans (Christen et al. 2014). Apart from changes in bone volume fraction, alterations in loading patterns can cause realignment of the trabecular structure towards a new axis of loading. In general, trabecular anisotropy is more pronounced in bones with a dominant loading direction than in bones subjected to more generalised loading patterns (Barak et al. 2011). To summarise, bone architecture, including local bone volume fraction and fabric orientation, serves as a biological record of mechanical loading history (Fischer et al. 1995).

The close interplay between bone morphology and mechanical function allows to estimate the average loading history from bone architecture and vice versa. Adequate estimation of *in vivo* loading conditions poses a relevant input for many biomedical applications such as patient-specific modelling of bone adaptation after surgical interventions or lifestyle changes, predictions of fracture healing processes, as well as implant design optimisation (Ghosh et al. 2022; Synek et al. 2019). Furthermore, such analyses can be used to detect pathological loading patterns in individuals or to gain information on the activity and motion patterns of extinct species (Synek et al. 2019). Estimation of *in vivo* loading conditions can be achieved through invasive methods, such as instrumented prostheses (Bergmann et al. 2016), or non-invasive methods, such as musculoskeletal modelling or inverse bone remodelling approaches using the finite element (FE) method (Bachmann et al. 2025). Musculoskeletal models are based on bone and muscle representations of the full body or entire limbs and estimate muscle and joint reaction forces corresponding to a defined motion (Sylvester et al. 2021). In contrast, inverse bone remodelling returns an overall habitual loading estimation based on bone structure and density information obtained by computed tomography (CT) images. In this approach, long-term loading conditions are estimated by identifying a set of forces and moments which minimise a stimulus-based objective function (Fischer et al. 1995; Christen et al. 2012; Bachmann et al. 2023a; Schenk and Zysset 2023). In the past, various biomechanical remodelling stimuli such as strain energy density, tissue stress or strain, as well as local damage accumulation, have been proposed (Huiskes et al. 1987; Beaupré et al. 1990; Cowin and Hegedus 1976; Prendergast and Taylor 1994; Calvo-Gallego et al. 2022). These mechanical quantities serve as surrogate measures for the complex underlying biological processes that govern mechanically induced bone remodelling. On a biological level, osteocytes embedded within the extracellular matrix are thought to sense mechanical stimuli such as lacunar-canalicular fluid flow, matrix strain or tissue damage and orchestrate the local bone remodelling process (Santos et al. 2009; Noble and Reeve 2000). Initial inverse bone remodelling algorithms were formulated for two-dimensional problems using density-based continuum FE models (Fischer et al. 1995). By using micro-FE instead of homogenised models, Christen et al. (2012) not only considered local bone density, but also bone microstructure in the load estimation of three-dimensional samples. This approach allowed to estimate plausible loading histories in computational studies of synthetic bone samples (Christen et al. 2013), murine vertebrae (Christen et al. 2012) and at the human hip (Synek and Pahr 2018). Recently, homogenised FE-based inverse bone remodelling has been proposed for three-dimensional problems in order to reduce the high amount of computational time required for such analyses (Schenk and Zysset 2023; Bachmann et al. 2023a, b, 2025). However, these models are density-driven and do not consider the local orientation of the trabecular structure in the remodelling algorithm. While local bone volume fraction accounts for 87-89 % of the variance in elastic properties of bone, the contribution of local fabric orientation (9-10 %) is not negligible (Maquer et al. 2015; Musy et al. 2017). The establishment of high-resolution peripheral quantitative computed tomography (HR-pQCT) in clinical workflows allows the acquisition of *in vivo* images with a sufficiently high resolution to capture bone microstructure (Cheung et al. 2013), thereby allowing the incorporation of fabric anisotropy in homogenised inverse bone remodelling. Zysset (2026) recently proposed a theoretical framework to determine an optimal tissue stress for a given local bone volume fraction and fabric orientation. However, this novel approach has so far only been described in the context of representative volume elements and has not yet been applied to larger biological bone samples. The present study aims to overcome the above-described limitations by including both local bone volume fraction and fabric orientation in homogenised inverse remodelling algorithms for the habitual load case estimation in bone on the example of the distal human tibia. Specifically, the impact of fabric anisotropy on habitual load case prediction, as well as the influence of St. Venant’s effect and load case complexity on the obtained results, is analysed. Further, the physiological plausibility of the estimated load cases is evaluated, and a bone safety factor relating the identified habitual loading to experimental yield forces is determined.

## Materials and methods

### Theoretical framework

#### Local inverse remodelling optimisation

Locally, an optimal stress state $\boldsymbol{\sigma }_\textrm{opt}$ maximising the stress intensity for a given target stimulus can be determined under consideration of the local bone volume fraction ρ and the fabric tensor $\textbf{M}$ for each representative volume element in an FE model. The local inverse remodelling approach used in this study follows the complementary free energy density-based optimisation procedure recently proposed by Zysset (2026), which assumes that bone adapts to minimise its mass while maintaining the mechanical stimulus related to habitual loading. Under habitual loading, the bone is in a state of homeostasis, and no changes in bone architecture occur. In this case, the modelling stimulus, which is represented by the local complementary free energy density $\Psi ^*$, is assumed to be equivalent to a setpoint stimulus $\Psi ^*_\textrm{set}$.

1 $$\begin{aligned} \Psi ^*(\boldsymbol{\sigma }_\textrm{opt},\rho ,{\textbf {M}}) = \frac{1}{2} \boldsymbol{\sigma }_\textrm{opt}:\mathbb {E}(\rho ,\textbf{M}) \boldsymbol{\sigma }_\textrm{opt} = \Psi ^*_\textrm{set} \end{aligned}$$

$\mathbb {E}(\rho ,\textbf{M})$ describes the density and fabric-dependent compliance tensor according to Zysset and Curnier (1995). In homogenised problems, the setpoint stimulus $\Psi ^*_\textrm{set}$ must be expressed at the continuum level, where the relationship between the homogenised and tissue-level stimulus can be expressed through a density-based homogenisation function f(ρ) (Bachmann et al. 2023a).

2 $$\begin{aligned} \Psi _\textrm{set}^* = \frac{f(\rho )}{f(1)} \Psi _\mathrm {set\_tissue}^* \end{aligned}$$

Normalisation by *f*(1) is required for homogenisation functions where f(1)≠1. In this study, the tissue-level setpoint energy density was set to $\Psi ^*_\mathrm {set\_tissue} = 0.037$ MPa, which is equivalent to a target tissue strain of 1800 $\mu \varepsilon$ for the material properties used in this study (*E* = 10490, ν = 0.2289). Depending on the rate and frequency of loading, daily tissue strain levels between 200 $\mu \varepsilon$ and 2000 $\mu \varepsilon$ are expected to maintain existing bone mass (Manske et al. 2009; Frost 2003; Bachmann et al. 2023a). Following the approach proposed by Zysset (2026), the optimal stress $\boldsymbol{\sigma }_\textrm{opt}$ is determined in two steps. First, the orientation of a normalised stress tensor $\boldsymbol{\hat{\sigma }}_\textrm{opt}$ is determined based on the local fabric orientation by minimising the complementary free energy density for ρ=1 under a stress normalisation constraint.

3 $$\begin{aligned} \boldsymbol{\hat{\sigma }}_\textrm{opt} \in \mathop {\arg \min }\limits _{\boldsymbol{\hat{\sigma }}\ \text {s.t.} \operatorname {tr}|\boldsymbol{\hat{\sigma }}|= 3} \Psi ^*(\boldsymbol{\hat{\sigma }},1,{\textbf {M}}) \end{aligned}$$

A normalised stress tensor $\boldsymbol{\hat{\sigma }}$ is defined as

4 $$\begin{aligned} \boldsymbol{\hat{\sigma }} = \frac{1}{\lambda _s} \boldsymbol{\sigma }, \quad \lambda _s = \sum _k^d \frac{|\sigma _k |}{d} \end{aligned}$$

with $\sigma _k$ being the stress eigenvalues and *d* being the number of dimensions considered in the problem. In three-dimensional problems, the resolution of the optimisation in Eq. (3) yields eight possible solutions, depending on the signs of the stress eigenvalues $\sigma _k$. However, as the complementary free energy density $\Psi ^*$ does not distinguish between tensile and compressive stress stimuli, solutions obtained from eigenvalue sets with opposite signs are equivalent (Zysset 2026). In the present study, the solution containing eigenvalues with three identical signs was defined as the normalised optimal stress $\hat{\boldsymbol{\sigma }}_\textrm{opt}$, since it corresponds to the global minimum of the normalised complementary free energy density for a given bone volume fraction ρ and fabric orientation $\textbf{M}$. A step-by-step guide for the determination of $\hat{\boldsymbol{\sigma }}_\textrm{opt}$ can be found in Zysset (2026). In a second step, the normalised stress tensor is scaled under consideration of the local bone fraction ρ, resulting in the homeostatic optimal stress state $\boldsymbol{\sigma }_\textrm{opt}$.

5 $$\begin{aligned} \boldsymbol{\sigma }_\textrm{opt} = \lambda _{s} \boldsymbol{\hat{\sigma }}_\textrm{opt} \end{aligned}$$

6 $$\begin{aligned} \lambda _{s} = \frac{f(\rho )}{\lambda _{\rho }}, \quad \lambda _{\rho } = \sqrt{\frac{\Psi ^*(\boldsymbol{\hat{\sigma }}_\textrm{opt}, 1, \textbf{M}) f(1)}{{\Psi }^*_\mathrm {set\_tissue}} } \end{aligned}$$

The hereby obtained stress tensor $\boldsymbol{\sigma }_\textrm{opt}$ is aligned with the fabric tensor $\textbf{M}$ and represents the highest stress state which is supported by the osseous structure without triggering bone remodelling, i.e. the associated remodelling stimulus $\Psi ^*$ is at the homeostatic level.

#### Global inverse remodelling optimisation

**Fig. 1:**
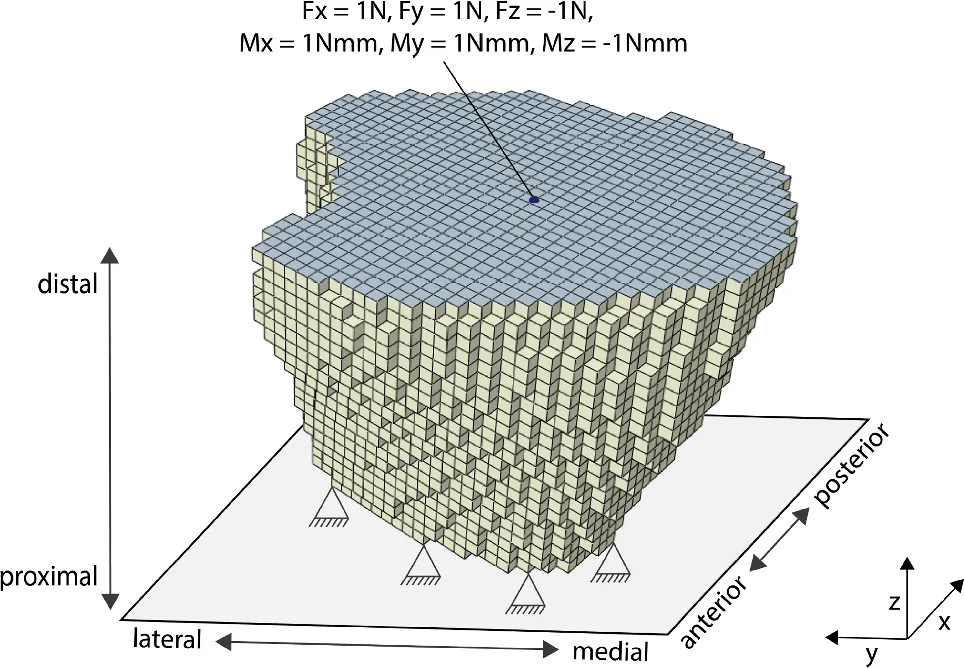
FE model of the distal human tibia oriented along the anatomical axes and corresponding boundary conditions

The average physiological loading history in a bone section can be approximated by a linear combination of three unit forces $\textbf{f}$ and three unit moments $\textbf{m}$ (Schenk and Zysset 2023) as illustrated in Figure 1. Similarly, the time-averaged stress state within the bone can be described as a linear combination of the stresses caused by these individual elementary forces and moments

7 $$\begin{aligned} \boldsymbol{\sigma }(\textbf{x}) = \sum _{i=1}^{n_\textrm{lc}}\alpha _i \boldsymbol{\sigma }_i(\textbf{x}) \end{aligned}$$

where $n_\textrm{lc}$ is the number of evaluated load cases. The ideal values for the scale factors α can be identified by minimising an objective function *OF*, which describes the normalised squared difference between the local stress state $\boldsymbol{\sigma }$ induced by the habitual load case and an ideal homeostatic stress state $\boldsymbol{\sigma }_\textrm{opt}$. As the local stress $\boldsymbol{\sigma }$ can be determined with the FE method, the objective function is discretised over the model mesh with $n_\textrm{el}$ being the number of finite elements.

8 $$\begin{aligned} OF = \frac{1}{V_\textrm{tot}}\sum _{n=1}^{n_\textrm{el}} \frac{1}{\boldsymbol{\sigma }_\textrm{opt}^n:\boldsymbol{\sigma }_\textrm{opt}^n} (\boldsymbol{\sigma }^n - \boldsymbol{\sigma }_\textrm{opt}^n) : (\boldsymbol{\sigma }^n - \boldsymbol{\sigma }_\textrm{opt}^n) V^n \end{aligned}$$

While the homeostatic stress state $\boldsymbol{\sigma }_\textrm{opt}$ is determined according to the local inverse remodelling optimisation as described in Sect. 2.1.1, the objective function in Eq. (8) considers the entire bone domain and leads to a global optimisation of the stress distribution within the bone. The objective function is normalised both with respect to total bone volume and homeostatic stress amplitude, which allows for an objective comparison between bones of different size, and ensures that the optimisation procedure is not dominated by highly anisotropic or dense regions within the bone. By substituting Eq. (7) into Eq. (8), we get the following optimisation problem.

9 $$\begin{aligned} & \mathop {\arg \min }\limits _{\alpha _i,\ 1 \le i \le n_{lc}} \frac{1}{V_\textrm{tot}} \sum _{n=1}^{n_\textrm{el}}\frac{V^n}{\boldsymbol{\sigma }_\textrm{opt}^n:\boldsymbol{\sigma }_\textrm{opt}^n}\left( \sum _{i=1}^{n_\textrm{lc}} \sum _{j=1}^{n_\textrm{lc}} \alpha _i \alpha _j \boldsymbol{\sigma }^n_i:\boldsymbol{\sigma }^n_j \right. \nonumber \\ & \quad \left. - 2 \sum _{i=1}^{n_\textrm{lc}} \alpha _i \boldsymbol{\sigma }^n_i: \boldsymbol{\sigma }_\textrm{opt}^n + \boldsymbol{\sigma }_\textrm{opt}^n : \boldsymbol{\sigma }_\textrm{opt}^n\right) \end{aligned}$$

The corresponding stationary points with respect to α are given by

10 $$\begin{aligned} \frac{dOF}{d\alpha _i}&= \frac{1}{V_\textrm{tot}} \sum _{n=1}^{n_\textrm{el}} \frac{ V^n}{\boldsymbol{\sigma }_\textrm{opt}^n:\boldsymbol{\sigma }_\textrm{opt}^n} \left( \sum _{j=1}^{n_\textrm{lc}} 2 \alpha _j \,\boldsymbol{\sigma }^n_i:\boldsymbol{\sigma }^n_j \right. \nonumber \\ & \quad \left.- 2 \boldsymbol{\sigma }^n_i : \boldsymbol{\sigma }_\textrm{opt}^n \right) ,\ 1 \le i \le n_\textrm{lc} \\&= 0 \end{aligned}$$

The optimal scale factors α can be identified by solving the resulting linear equation system of the form $A_{ij}\alpha _j + b_i = 0$ with

11 $$\begin{aligned} A_{ij} = \frac{2}{V_\textrm{tot}} \sum _{n=1}^{n_\textrm{el}} \frac{V^n}{\boldsymbol{\sigma }_\textrm{opt}^n:\boldsymbol{\sigma }_\textrm{opt}^n} \boldsymbol{\sigma }^n_i:\boldsymbol{\sigma }^n_j \end{aligned}$$

and

12 $$\begin{aligned} b_i = \frac{2}{V_\textrm{tot}} \sum _{n=1}^{n_\textrm{el}} \frac{V^n}{\boldsymbol{\sigma }_\textrm{opt}^n:\boldsymbol{\sigma }_\textrm{opt}^n} \boldsymbol{\sigma }^n_i : \boldsymbol{\sigma }_\textrm{opt}^n \end{aligned}$$

### Finite element model generation

Donor-specific homogenised finite element models of 25 human distal tibia sections from twelve female (age: 84 ± 10 years) and four male (age: 77 ± 9 years) donors were generated following the procedure presented in Simon et al. (2024). All samples were obtained from the Division of Anatomy of the Medical University of Vienna, Austria, with the approval of the ethics committee of the Medical University Vienna (no 2339/2019) and originated from individuals who voluntarily donated their bodies for anatomical education and research purposes. In brief, linear hexahedral FE meshes with a maximum element size of 1.27 mm were built from segmented and calibrated micro-CT images. One sample was excluded from the original dataset due to inaccurate segmentation results. Element-specific bone volume fraction ρ and fabric orientation $\textbf{M}$ were determined from the high-resolution images as described in Schenk et al. (2022). For the present study, all FE meshes were reoriented such that the sample’s anterior-posterior, medio-lateral and proximal-distal axes were aligned with the global* x*,* y* and* z* axes, respectively (Fig. 1). Right samples were mirrored in the sagittal plane to eliminate differences in loading direction between left and right samples. A detailed description of the reorientation algorithm can be found in Appendix 1. As the present study investigates habitual bone loading in the physiological regime only, an anisotropic linear elastic constitutive model (Zysset and Curnier 1995) without damage or plasticity was used for the computational simulations. Young’s modulus (*E* = 10490 MPa) and Poisson ratio (ν = 0.2289) were defined based on experimental tests and computational homogenisation studies (Wolfram et al. 2010; Panyasantisuk et al. 2015), and the corresponding stiffness tensor was scaled with the density function f(ρ) in each element (Appendix 2). All nodes in the sample’s most proximal layer were fully encastered, while all nodes in the most distal plane were kinematically coupled to a reference node placed in the corresponding plane, which was vertically aligned with the mesh centroid (Fig. 1). Six canonical unit loads ($F_x$ = 1N, $F_y$ = 1N, $F_z$ = -1N, $M_x$ = 1Nmm, $M_y$ = 1Nmm, $M_z$ = -1Nmm) were imposed in individual simulations on this reference node. All FE simulations were run on Abaqus Standard 2021.HF6 (Simulia, Dassault Systémes, Paris, France) using a desktop computer with an Intel Core i7-11700F processor (Intel Corporation, Santa Clara, California, U.S.) using eight CPU cores.

### Load case estimation and evaluation

The theoretical framework presented in Sect. 2.1 was applied to the finite element models presented in Sect. 2.2. The inverse bone remodelling pipeline was implemented in Python 3.13.2 (Python Software Foundation, https://www.python.org/) and is publicly available on zenodo (Gerber and Zysset 2026). The FE simulation results corresponding to the six unit load cases were imported to Python with the software ODB2VTK (Shen et al. 2023). Subsequently, the optimal stress state for each element was determined following Sect. 2.1.1 and optimal scale factors for the load case estimation were identified by solving the linear equation system defined by Eqs. (11, 12). In total, seven variations of the global inverse remodelling optimisation approach were analysed in this study. To avoid any influence of the St. Venant effect on the results, the two most proximal and distal element layers were removed from the optimisation domain, thereby preventing unphysiological stress states close to the boundary layer from influencing the optimisation results. Similarly, elements containing more cortical than trabecular bone in the segmentation mask were filtered out to evaluate the influence of the cortical shell on the remodelling outcomes. Local fabric anisotropy was either considered in both the FE simulations and the remodelling optimisation, the remodelling only, or fully substituted by an isotropic material in both cases. A detailed analysis of the local variation of the objective function along the sample’s longitudinal axis was performed for the full model including anisotropy, cortical bone and boundary layer elements. To quantify the influence of load case complexity on the objective function value, this analysis was conducted using optimisation results that considered the full set of forces and moments, forces only, and a pure axial force. Table 1 provides an overview of the different optimisation approaches analysed in this study and their associated features. The standard model (STD), including anisotropy, cortical bone, and no boundary layer elements, serves as a reference model for this study. Differences in optimised forces, moments, and objective function values across optimisation types were formally evaluated with the Friedman test due to non-normality of the corresponding distributions. Post-hoc comparisons were performed for significant outcome variables with respect to the standard model (STD) using a Bonferroni-corrected paired sign test. The statistical analysis was done in R 4.5.1 (R Core Team 2025). Additionally, a bone safety factor was calculated as the ratio of the optimised axial force estimated from the standard model (STD) and the yield force obtained from corresponding compressive experiments (Schenk et al. 2022).

**Table 1:** Optimisation types used to investigate the impact of fabric anisotropy, St. Venant effect, cortical shell and load case complexity on habitual load case estimation in the distal human tibia

Optimisation type	Model name	Anisotropic FE	Anisotropic optimisation	Cortex included	Boundary layers excluded	Forces and moments	Forces only	$F_z$ only
Standard	STD	×	×	×	×	×		
No cortex	NO-CORT	×	×		×	×		
Isotropic optimisation	ISO-OPT	×		×	×	×		
Fully isotropic	ISO-FULL			×	×	×		
Boundary layer inclusion	BL-INC	×	×	×		×		
Forces only	F-XYZ	×	×	×			×	
$F_z$ only	F-Z	×	×	×				×

## Results

### Optimised forces and moments

**Fig. 2:**
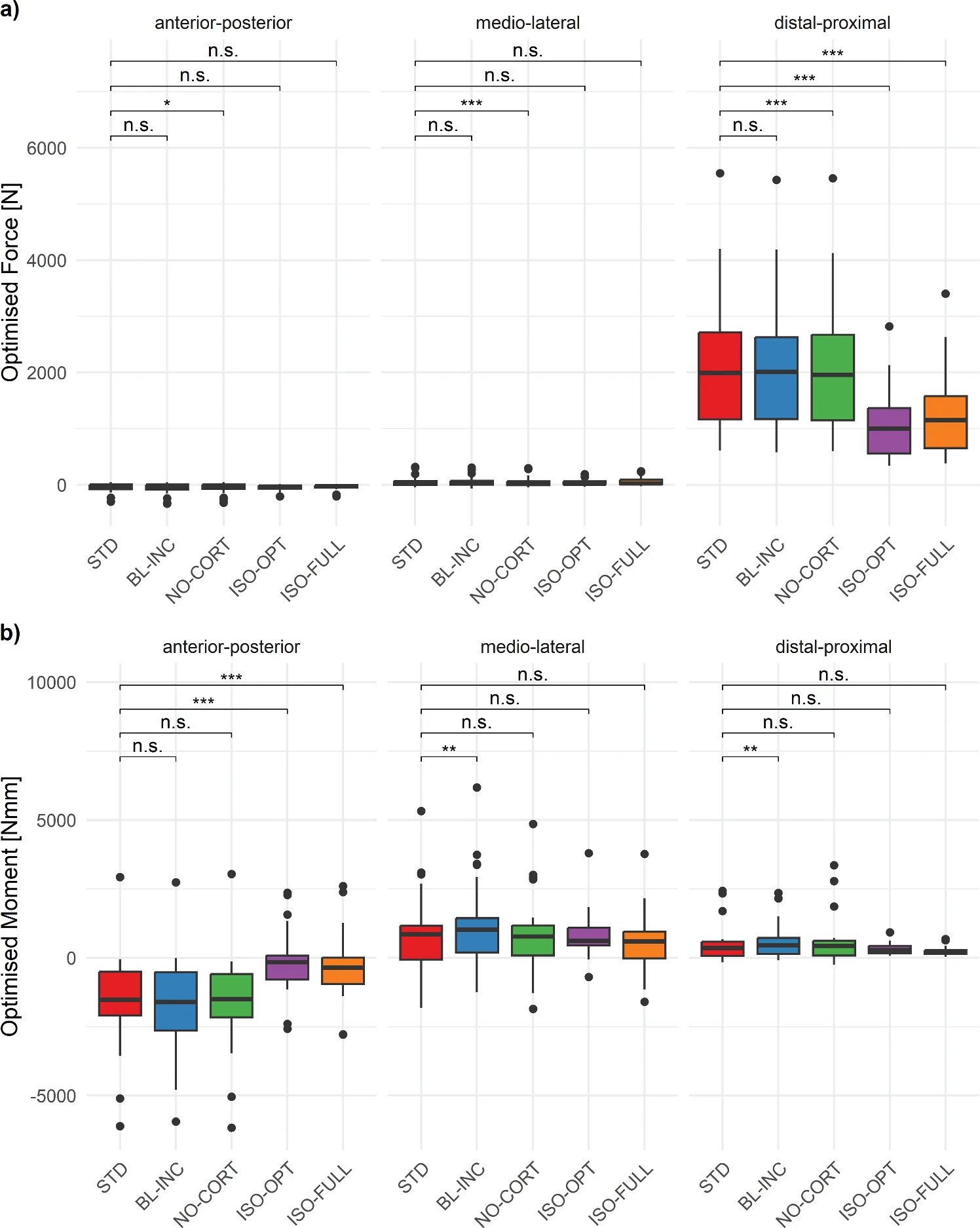
**a** Optimised forces and **b** optimised moments for different inverse remodelling approaches. Significance levels (n.s.: not significant, *p < 0.05, **p < 0.01, ***p < 0.001) are reported for Bonferroni corrected paired sign tests

Optimised forces and moments for optimisation approaches with full load case complexity are presented in Figure 2. Corresponding results for optimisations with reduced load case complexity are provided in Appendix 3 for completeness. In all cases, the dominant force component corresponded to a compressive force acting along the longitudinal axis of the bone. Assuming a mass of 80 kg for male donors and 67 kg for female donors (Stival et al. 2022), this axial load corresponded to 3.08 ± 2.23 times body weight for the standard model (STD). All shear forces along the anterior-posterior and medio-lateral axes were close to zero. Irrespective of the optimisation approach, negative moments about the anterior-posterior axis, and positive moments about the medio-lateral and distal-proximal axis were found. Statistical inference results for the post-hoc tests are reported in Figure 2 for all outcome variables, since the corresponding Friedman tests were positive (*p* < 0.05). Although some significant differences between models without cortex (NO-CORT) and the standard model (STD) as well as between models including boundary layer elements (BL-INC) and the standard model (STD) were found, the effect size when varying these features was small, and the estimated forces and moments remained largely unaffected. In contrast, fully (ISO-FULL) or partially (ISO-OPT) isotropic remodelling approaches resulted in a relevant reduction in the estimated axial force and anterior-posterior axis moment. By comparing the axial compressive force from the standard model (STD) and the corresponding yield force determined by compressive experiments, an average safety factor of 3.62 ± 0.72 was identified.

### Objective function

**Fig. 3:**
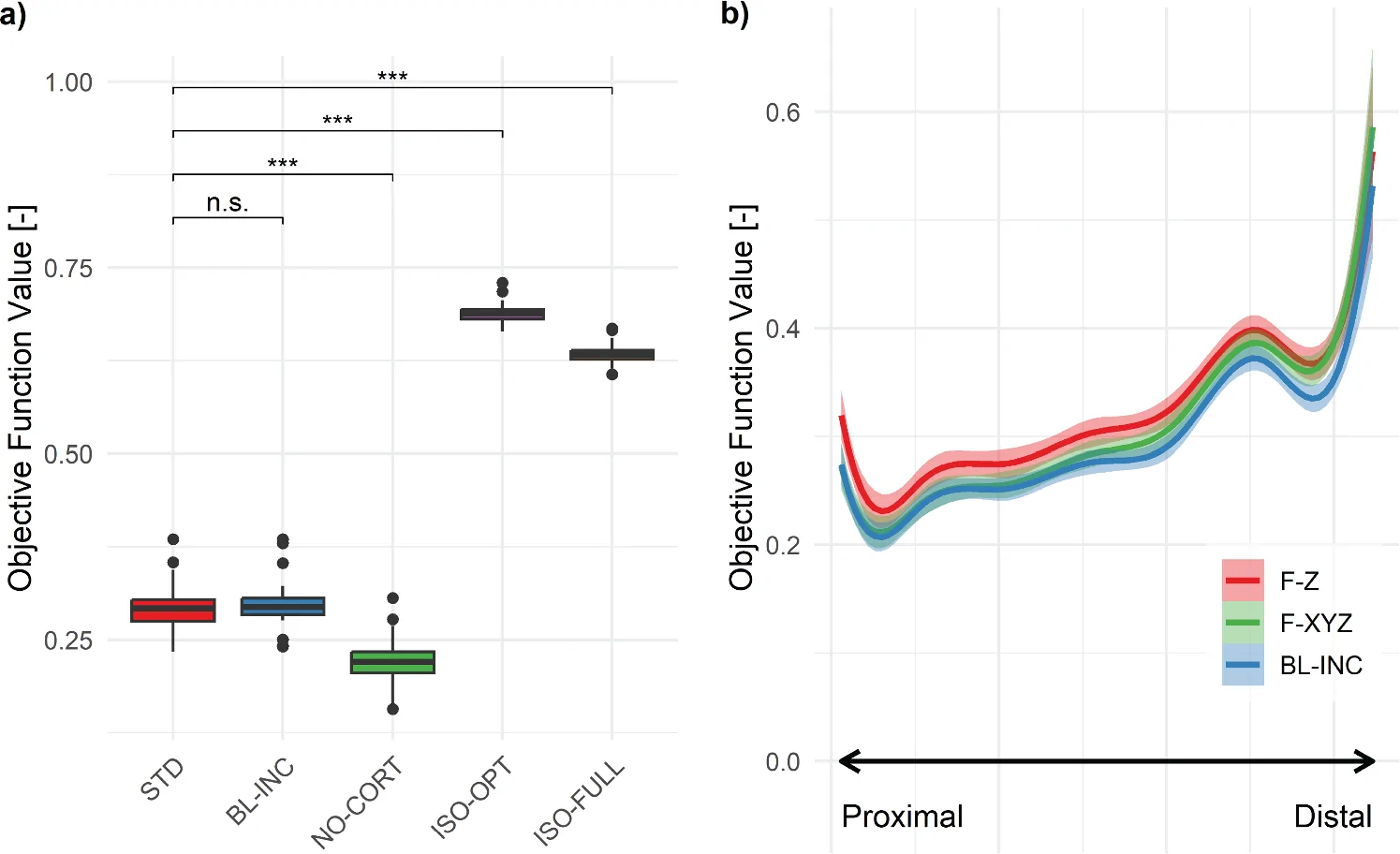
**a** Objective function values for different inverse remodelling approaches. Significance levels (n.s.: not significant, *p < 0.05, **p < 0.01, ***p < 0.001) are reported for Bonferroni corrected paired sign tests. **b** Change in average objective function value along the sample’s longitudinal axis for models including boundary layer elements (BL-INC) and varying load case complexity *(F*-*Z*: axial force only, *F*-*XYZ*: all forces, BL-INC: all forces and moments)

**Fig. 4:**
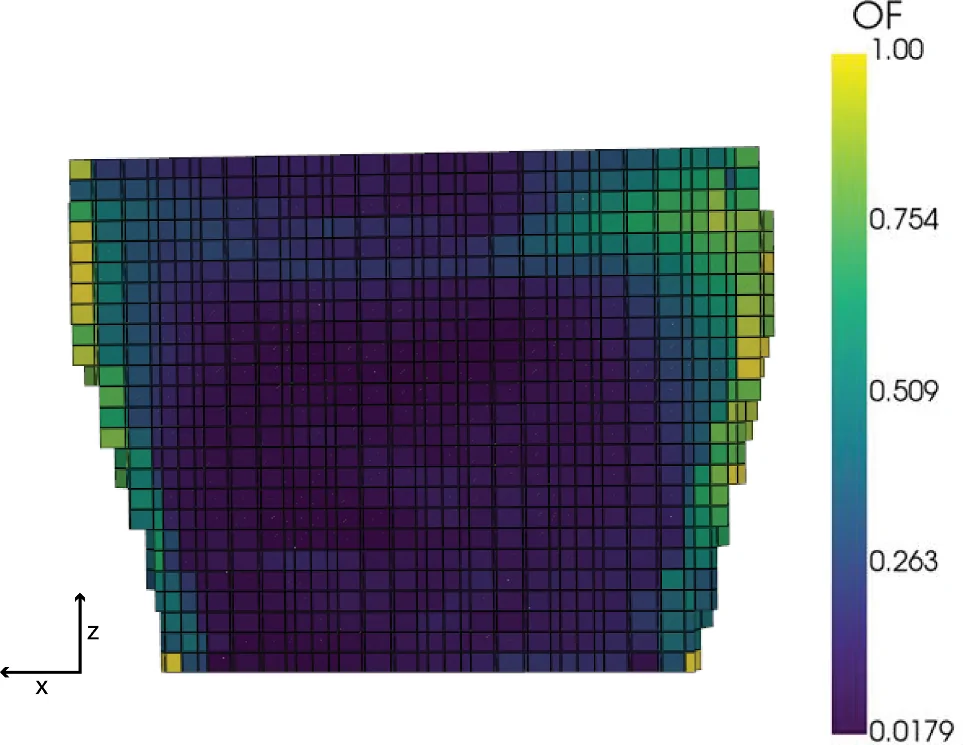
Sagittal view of the objective function distribution in a typical distal tibia section under optimised loading conditions. Objective function values were cieled at 1 for better visualisation

Objective function values and corresponding inference results from post-hoc tests are presented in Figure 3. Removing predominantly cortical elements from the analysis volume (NO-CORT) led to a reduction of the objective function by 25 %, while omission of anisotropic properties (ISO-FULL, ISO-OPT) increased the value approximately by a factor of two. For models including boundary layer elements (BL-INC), a steep increase in the objective function at the most proximal and distal boundary layers was observed (Fig. 3). Additionally, a general increase in the objective function towards the distal portion of the tibia section and in the vicinity of the cortical shell was found (Fig. 4). Simplifications of the considered load case by removing force and moment contributions consistently led to an increase in the objective function. The curves displayed in Figure 3b represent B-splines of degree eight with the corresponding standard error fitted to the local objective function value distribution. An overlay of each fitted spline with the underlying original data is presented in Appendix 3. Accounting for anterior–posterior and medio–lateral force components led to greater reductions of the objective function in the proximal region of the sample, whereas the inclusion of moments predominantly affected the distal regions of the tibia section. The overall objective function values were significantly higher for optimisations with reduced load case complexity (*p* < 0.01) than for the model including both forces and moments in the load case representation.

## Discussion

### Physiological load case

**Fig. 5:**
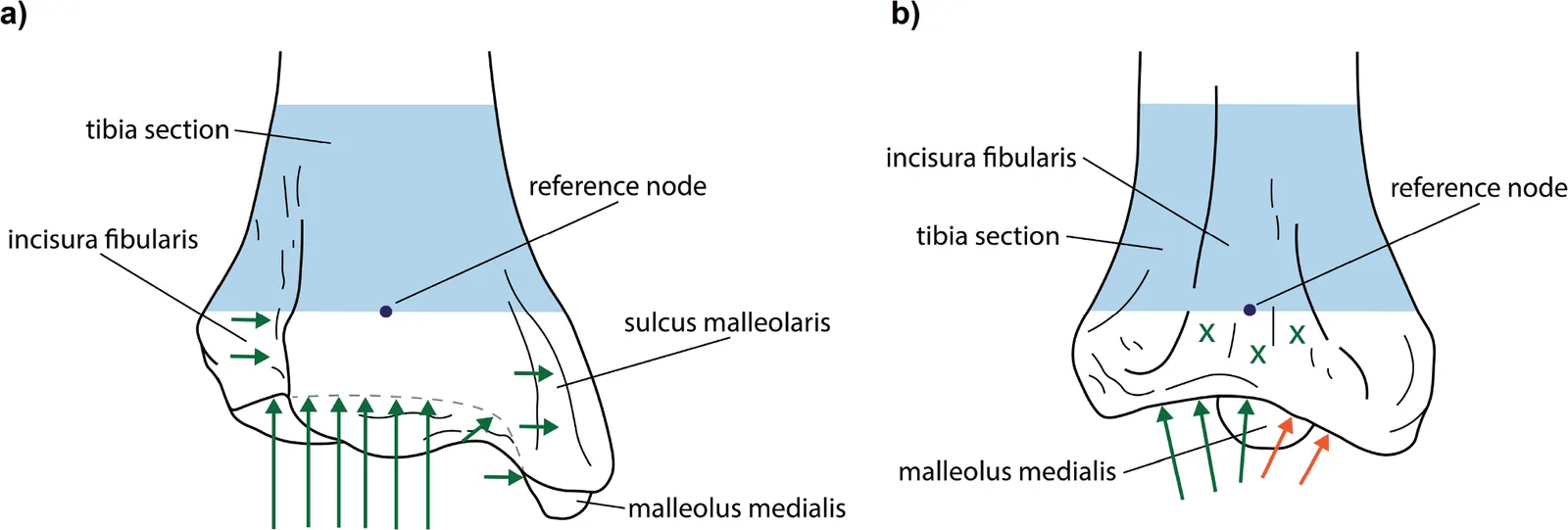
Schematic analysis of the forces acting on the human distal tibia during gait in healthy individuals, shown from **a** posterior and **b** lateral view. The forces present during heel-strike are highlighted in orange for the lateral view

The optimised forces and moments obtained with the anisotropic inverse remodelling algorithm presented in this study represent a realistic habitual loading scenario for human distal tibia sections. In everyday life, the tibia is mainly loaded during bipedal ambulatory motion such as walking, running or stair climbing. Joint reaction forces at the talocrural joint are predominantly oriented along the longitudinal axis of the tibia (Unnikrishnan et al. 2018) and peak shortly before heel-off during walking (Fraysse et al. 2016; Valente et al. 2014). The inverse bone remodelling algorithm presented in this study indeed identified the axial compressive force as the dominant load component. Nevertheless, considering shear forces and moments in the load case estimation resulted in a significant reduction of the objective function, suggesting that the inclusion of these load components, albeit small in magnitude, may still be relevant to adequately reflect physiological loading conditions. Small forces in the transversal plane can stem from pressure on articulating surfaces such as the *malleolus medialis* or the *incisura fibularis* as well as from passive stabilisation through the ligament apparatus, while moments are induced by off-axis joint reaction forces relative to the computational model’s reference node. In healthy individuals, the contact area between the talar dome and the tibial plafond is shifted laterally (Rajaeirad et al. 2024), inducing a negative moment about the anterior-posterior axis of the foot. Further, densitometric and microstructural analysis of the ultradistal tibia revealed higher bone mineral density as well as increased trabecular number and thickness in the posterior region of the tibia when compared to the anterior portion of the bone (Unnikrishnan et al. 2018; Lai et al. 2005). During the stance phase of walking, the centre of pressure shifts from a posterior position to an anterior position and back (Son et al. 2023). Although peak contact force is reached in the anterior region of the talocrural joint surface (Son et al. 2023), the dynamic nature of the force impact at heel strike may trigger a higher osteogenic response in the posterior distal tibia (Lai et al. 2005; Lanyon and Rubin 1984). These findings are reflected by a positive moment about the medio-lateral tibia axis in our inverse remodelling optimisation. A small positive moment about the distal-proximal tibia axis can be induced by the tendons of *m. tibialis posterior* and *m. flexor digitorum longus* running through the *sulcus malleolaris tibialis* located on the posteromedial surface of the tibia. A schematic representation of the forces acting on the distal tibia during gait can be found in Figure 5. While the optimised forces and moments are in good agreement with loading conditions in the dominant load case for the distal tibia, it should be noted that results obtained from inverse remodelling generally represent an average loading history acting on a bone and do not correspond to a single load case.

Peak joint reaction forces at the ankle predicted by musculoskeletal models range from four to six times body weight for physiological gait analysis (Fraysse et al. 2016; Valente et al. 2014; Brockett and Chapman 2016). In the present study, the average axial compressive load (3.08 x body weight) was slightly lower than these estimates. However, no information was available about the donor’s size, weight, activity levels or health status, and body weight-converted load cases should not be interpreted as exact predictions in this study. Additionally, loading conditions estimated with musculoskeletal modelling and inverse bone remodelling may differ in magnitude since the latter considers long-term averaged loading conditions instead of forces corresponding to a specific motion, or due to inaccurate set-point stimulus calibration. Nevertheless, the order of magnitude of the predicted axial force is within a reasonable range for an elderly population. Furthermore, the bone safety factor identified in this study (SF = 3.62) is quantitatively close to literature values, where a safety factor of three for load-bearing long bones was suggested (Turner 1992). The existence of such a safety factor prevents the failure of bone during strenuous activities, where peak loads beyond average daily activity levels are reached.

### Fabric inclusion

Including fabric anisotropy in inverse bone remodelling significantly affected the resultant force and moment estimates in both magnitude and orientation. Specifically, the axial compressive force and the moment about the anterior-posterior axis were strongly increased when the microstructural orientation was considered in the optimisation algorithm, while the other load case components remained largely unchanged. Hence, by aligning the optimised load case with the bone’s trabecular structure, the bone samples tolerate higher load magnitudes for a given setpoint. Strongly reduced objective function values in anisotropic optimisation procedures further confirm that the inclusion of fabric leads to improved agreement between predicted load cases and the bone architecture for the selected modelling criterion. Importantly, fabric anisotropy must be considered both in the constitutive model of the original FE simulation as well as in the inverse remodelling optimisation. When applying an isotropic optimisation approach to anisotropic simulation outputs, the above-described effects are not present.

### Boundary conditions and St. Venant effect

In FE modelling, local stress distributions close to the model boundary can be strongly influenced by the exact implementation of boundary conditions. However, according to St. Venant’s principle, stresses further away from the model surface are not affected by variations in statically equivalent boundary conditions. In the present study, a sudden increase in objective function values was found in the most proximal and distal element layers. While the predicted load case leads to good agreement between the actual stress state $\boldsymbol{\sigma }$ and the fabric and density-derived ideal stress state $\boldsymbol{\sigma }_\textrm{opt}$ in the sample’s core, high differences were found in elements located in the boundary layers. Such local discrepancies potentially arise due to the approximation of the habitual load case by a linear combination of three base forces and moments applied to a local reference node in bone sections, whereas *in vivo* loading conditions represent a smooth distribution of forces applied at the articular surfaces of the joint. Similarly, an increasing trend in objective function values was observed from proximal to distal sample sections. The distal portion of the analysed tibia section is located more closely to the joint surface, which is expected to experience a higher load case variability than the proximal part located towards the diaphyseal region. Nevertheless, neither the average objective function value nor the force and moment estimations were strongly affected by the exclusion of boundary layer elements in the load estimation algorithm, despite relatively high local stress discrepancies in these regions. Therefore, the consideration of the St. Venant effect in homogenised inverse bone remodelling for distal tibia sections is primarily relevant when local stress distributions are of interest.

### Cortical bone

High objective function levels were found in the proximity of the cortical shell. As the objective function values within the mesh volume are relatively low and homogeneously distributed, these findings might indicate that the remodelling theory presented in this study is predominantly suitable for trabecular bone. However, many elements directly on the model surface exhibit relatively low bone volume fractions due to partial volume effects. The inaccurate representation of the cortical shell in voxel-based meshes provides an alternative explanation for the reduced agreement between optimised and ideal stress states in these models. Further investigations are needed to better understand this phenomenon. Although the removal of the cortical shell from the analysis volume led to a significant reduction in the objective function, the predicted forces and moments remained largely unaffected.

### Limitations

The inverse remodelling theory, which builds the foundation of this study, relies upon several assumptions. In the present study, the target tissue remodelling stimulus used to identify the local optimal stress state was based upon the complementary free energy density, which aggregates complex biological remodelling processes into a simple scalar value. Systemic factors such as mineral homeostasis or hormonal levels, which influence bone resorption and formation in a non-targeted way and interfere with an exclusively load-driven bone adaptation process (Kenkre and Bassett 2018; Christen et al. 2012), are not included in our approach but could be considered by adjusting the setpoint stimulus $\Psi ^*_\textrm{set}$. Further, the quantitative value of the target remodelling stimulus directly determines the amplitude of the optimised load case identified by the herein presented inverse remodelling algorithm, whereby the predicted load cases scale with the square root of the setpoint stimulus. Although this value was determined based on literature estimates, thoroughly controlled in vivo studies are required to calibrate our model and experimentally validate the predicted load cases. On the material level, the complementary free energy density minimum is reached for stress states with purely compressive stress eigenvalues. While 63 % of all elements reached such a stress state after load case optimisation, tensile principal stresses due to Poisson coupling were observed in elements located towards the sample’s surface (see Appendix 4). However, such deviations from the optimal local stress state on the structural level are to be expected, since bone must sustain a variety of loading scenarios in everyday life, and cannot be perfectly optimised towards a single load case. Finally, it is assumed that the habitual load case identified by our inverse remodelling scheme represents long-term average loading conditions. However, the model currently does not consider any time scales involved in the biological aspects of bone remodelling, nor temporal information related to the loading process, such as the rate and frequency of load application.

## Conclusion

In this study, we successfully included fabric anisotropy into homogenised inverse bone remodelling algorithms for the estimation of long-term habitual loading conditions in bone. The impact of microstructural orientation, St. Venant effect and load case complexity on the optimisation outcomes was analysed using a sample dataset of 24 human distal tibia sections. Our findings suggest that even for bones primarily experiencing uniaxial compression, the inclusion of additional load components such as transversal forces or moments has a beneficial effect on optimisation quality. Although the exclusion of boundary layer elements did not affect global optimisation outcomes in this study, elements directly subjected to boundary constraints contained high objective function values and should be treated with caution for local analyses. Accounting for both local bone volume fraction and microstructural orientation in the inverse remodelling scheme resulted in physiologically plausible load cases characterised by an increase in the predicted load magnitude and a reduction in objective function values. While these findings confirm the relevance of the microstructural orientation in inverse bone remodelling algorithms for the selected complementary free energy density-based remodelling criterion, future research activities must include model calibration and validation based on longitudinal *in vivo* experiments to assess the physiological accuracy of the estimated load cases. Additionally, advanced representations of the cortical shell by a smooth two-phase model and boundary conditions that account for local force distributions may lead to further improvement in load case predictions using homogenised finite element models. The herein presented inverse remodelling algorithm for bone provides a valuable tool for bone research and the development of medical implants. Furthermore, HR-pQCT images resolving the trabecular structure allow the translation of the remodelling scheme into clinical workflows, where the determination of long-term *in vivo* loading patterns may provide additional input for personalised treatment strategies.

## Figures and Tables

**Fig. 6:**
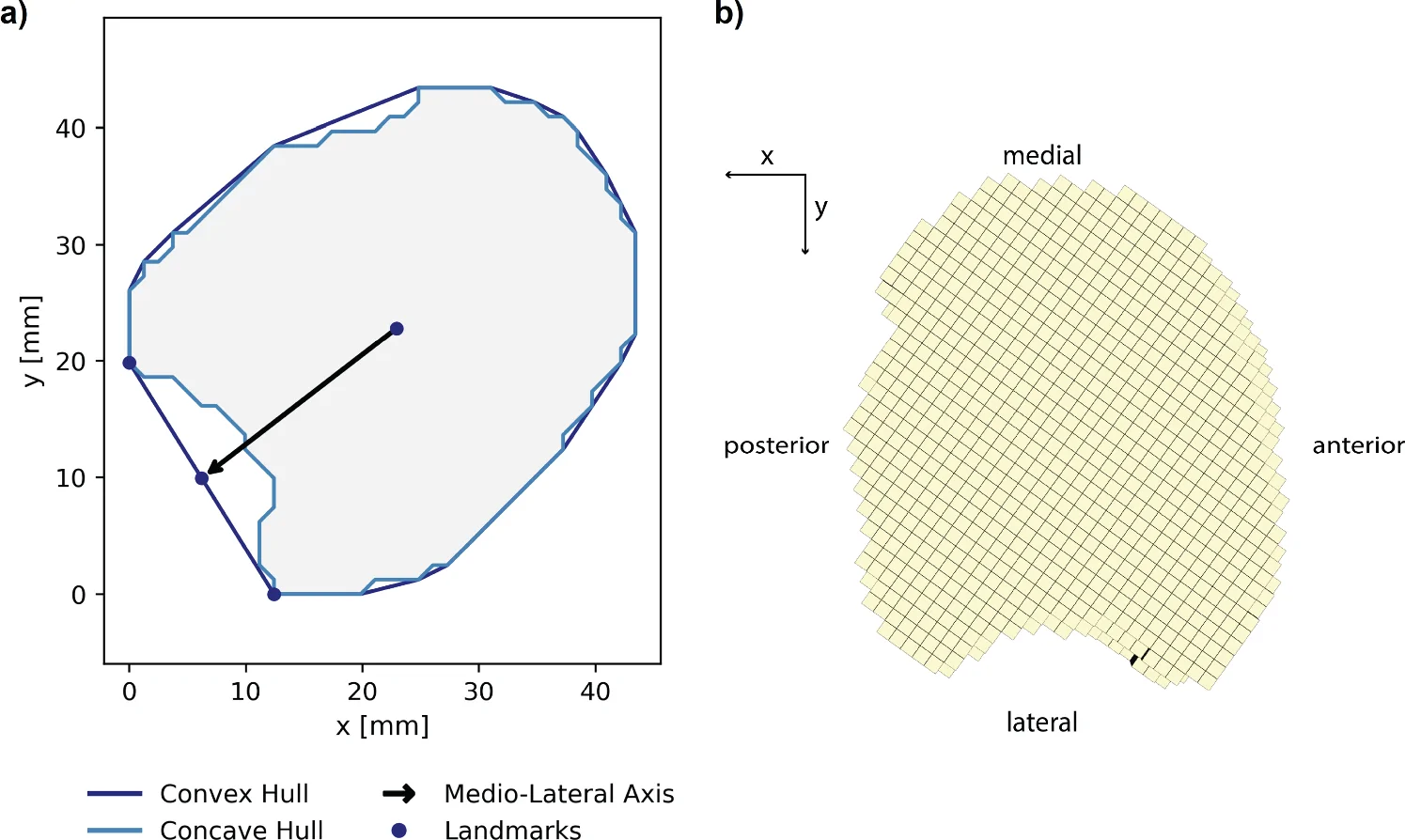
**a** Landmarks for tibia reorientation in the most distal plane (*A*: posterior tubercle, *B*: anterior tubercle, *C*: point cloud centroid, *M*: midpoint of *incisura fibularis tibiae*). **b** Transversal view of the mesh after realignment

**Fig. 7:**
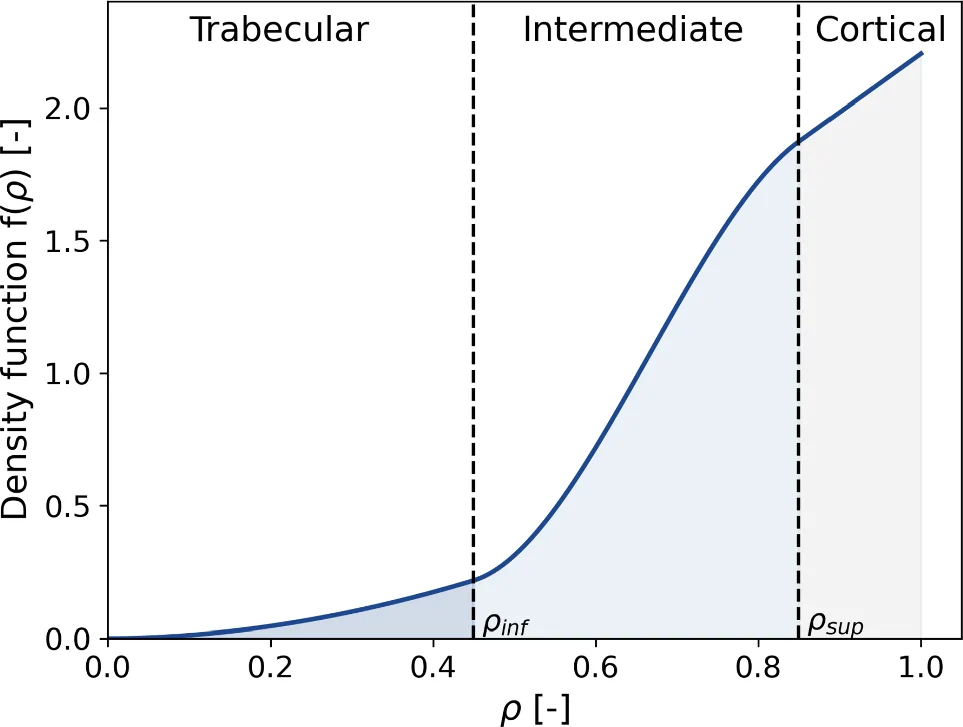
Density function f(ρ) for trabecular bone, cortical bone and intermediate elements. Figure reproduced from Gerber et al. (2025) published under a Creative Commons CC-BY license

**Fig. 8:**
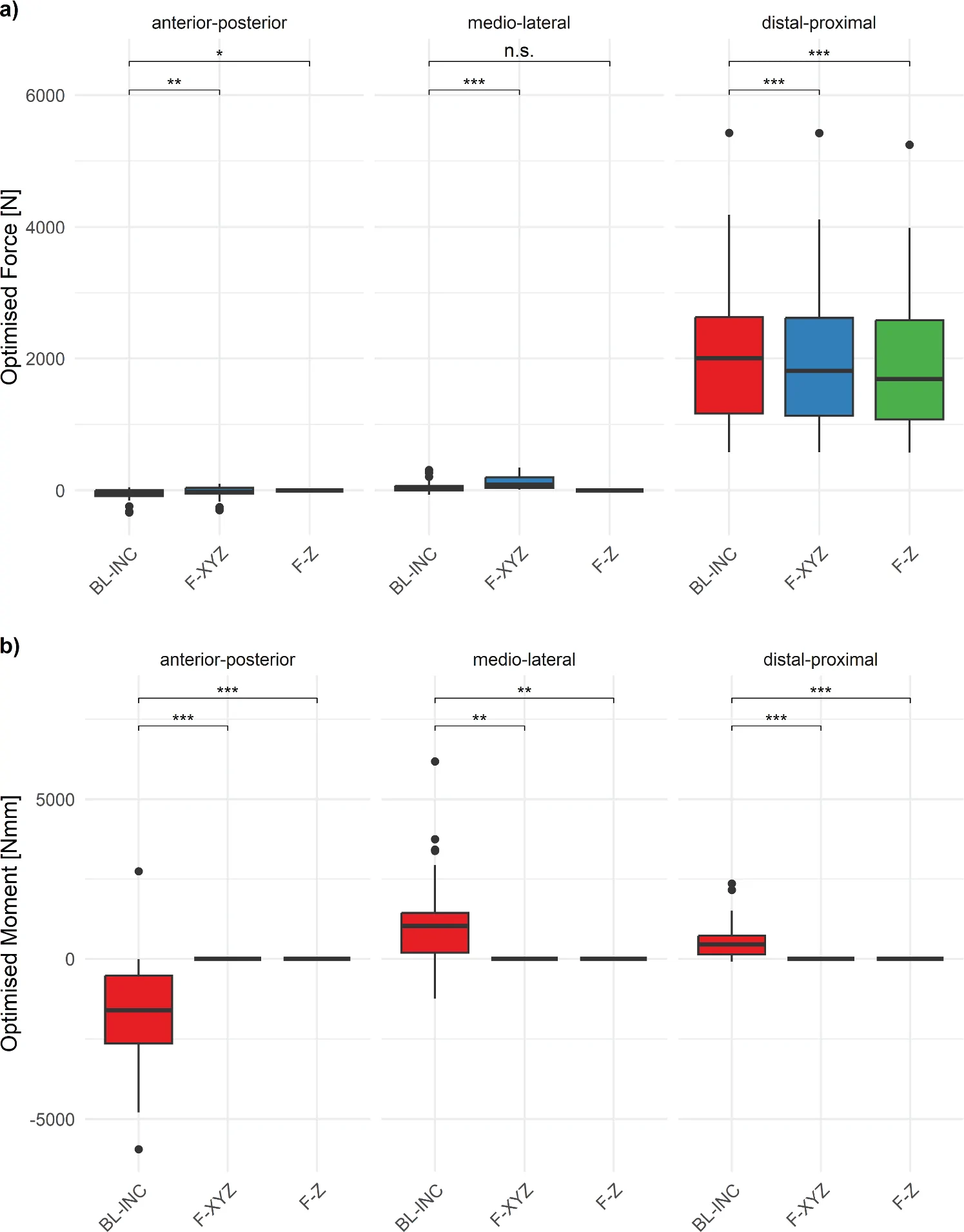
**a** Optimised forces and **b** optimised moments for inverse remodelling approaches with varying model complexity (*F*-*Z*: axial force only, *F*-*XYZ*: all forces, BL-INC: all forces and moments). Significance levels (n.s.: not significant, *p < 0.05, **p < 0.01, ***p < 0.001) are reported for Bonferroni corrected paired sign tests.

**Fig. 9:**
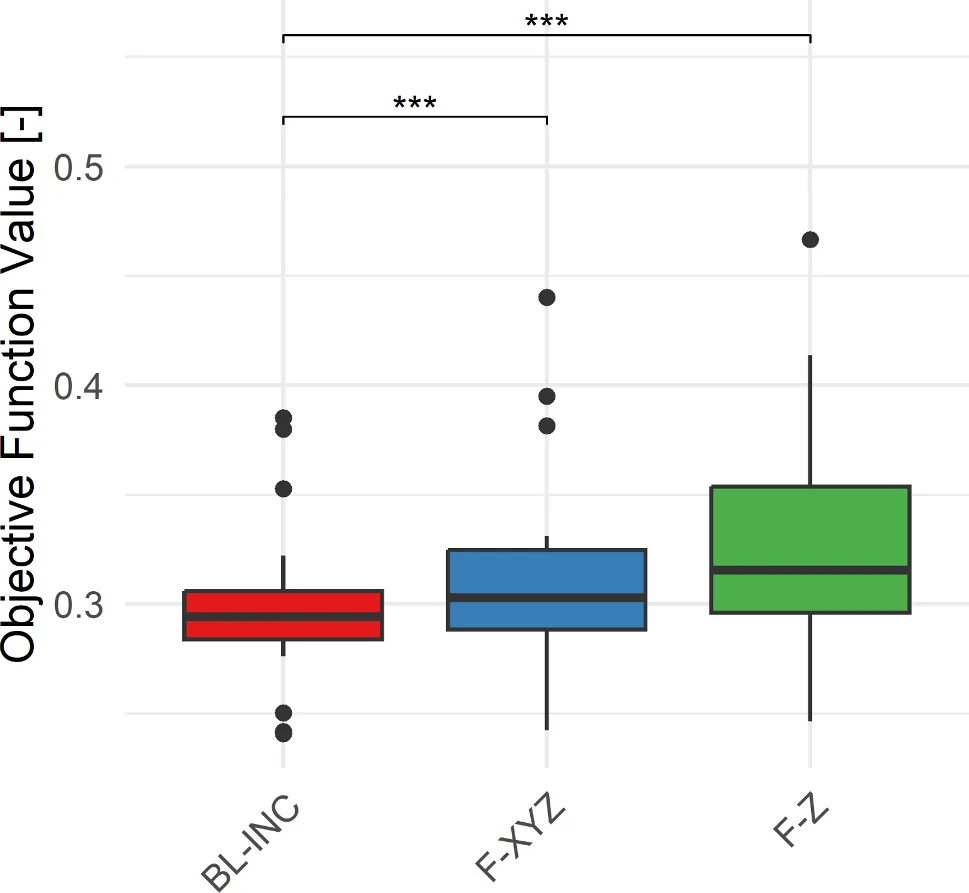
Objective function values for inverse remodelling approaches with varying model complexity (*F*-*Z*: axial force only, *F*-*XYZ*: all forces, BL-INC: all forces and moments). Significance levels (n.s.: not significant, *p < 0.05, **p < 0.01, ***p < 0.001) are reported for Bonferroni corrected paired sign tests

**Fig. 10:**
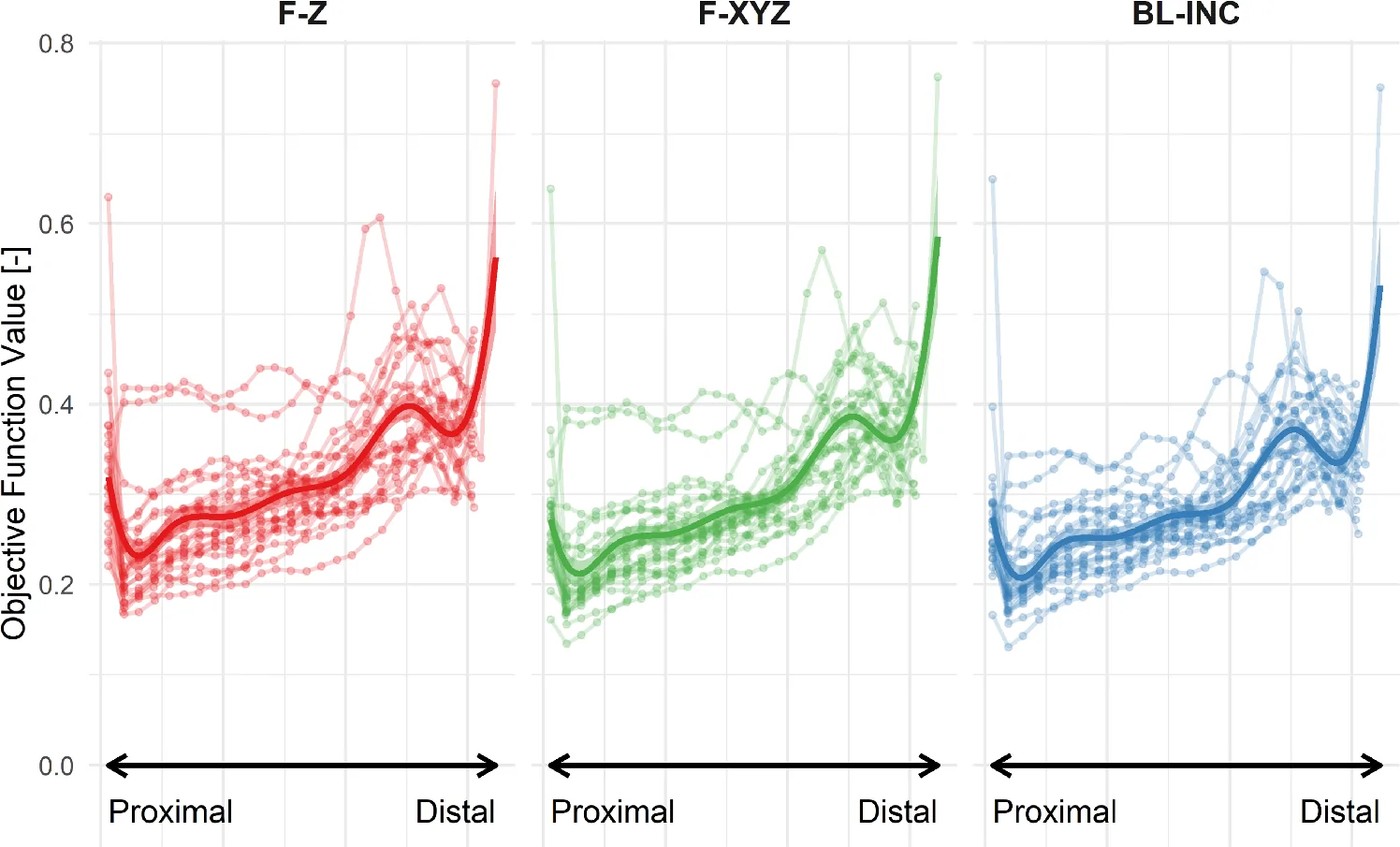
B-spline fits and underlying original data for local objective function values obtained from inverse remodelling optimisations with varying load case complexity (*F*-*Z*: axial force only, *F*-*XYZ*: all forces, BL-INC: all forces and moments)

**Fig. 11:**
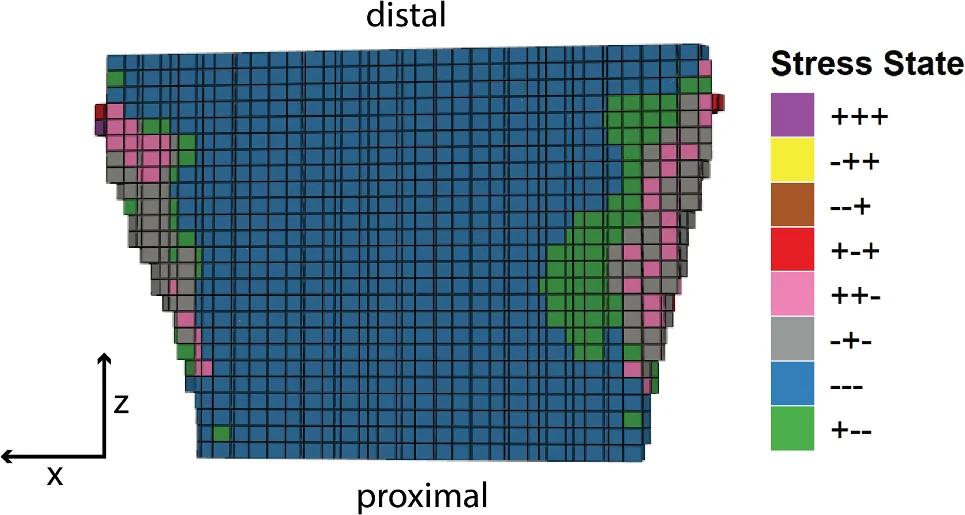
Sagittal view of the local stress state distribution in a typical distal tibia section under optimised loading conditions. Stress states were defined by the signs of the elemental principal stresses sorted in increasing order of their corresponding absolute values

## Data Availability

The code for FE model generation, inverse bone remodelling and data processing is shared open source on zenodo (https://doi.org/10.5281/zenodo.20280518). The data used in this study are available upon reasonable request.

## Appendix 1: Reorientation algorithm

Determination of the joint coordinate system of the ankle complex according to the International Society of Biomechanics (ISB) requires consideration of anatomical landmarks on the tibia and the fibula (Wu et al. 2002). However, donor-specific anatomical information was only available for distal tibia sections in this project. Therefore, a custom-made reorientation algorithm was used to align the tibia samples with the global coordinate axes. In the original FE meshes, the longitudinal axis naturally coincided with the global z-axis, as the micro-CT scans were taken along the longitudinal axis of the tibia sections. On the other hand, the global x- and y-axis were randomly oriented in the transversal plane. Sample realignment was performed with landmarks detected in the most distal plane of the tibia section (Fig. 6). The anatomical medio-lateral axis was defined as the connecting line between the midpoint of the fibular notch (*incisura fibularis tibiae*) and the centroid of the point cloud formed by all distal layer nodes. The anterior and posterior margins of the *incisura fibularis* were automatically identified as the intersection points of the concave and convex hull of the point cloud, where the largest area enclosed by the two contours marks the *incisura fibularis*. Next, the mesh was rotated in the transversal plane such that the medio-lateral axis was aligned with the global y-axis. As the anatomical coordinate system was chosen as an orthogonal right-handed coordinate system, the sample’s anterior-posterior axis corresponded to the global x-axis by definition.

## Appendix 2: Density function

The density function f(ρ) scales the local stiffness tensor $\mathbb {S}(\rho ,\textbf{M})$ with the local bone volume fraction ρ. While this relationship is commonly formulated as an exponential power law for trabecular bone (Helgason et al. 2008; Oftadeh et al. 2015), a linear relationship can be assumed for cortical bone (Cai et al. 2019). In the present study, a density function f(ρ) distinguishing between trabecular elements ($\rho \le 0.45$), cortical elements ($\rho \ge 0.85$), and homogenised elements with intermediate bone volume fractions was used (Figure 7) (Gerber et al. 2025).

$$\begin{aligned} f(\rho ) = {\left\{ \begin{array}{ll} \rho ^k & \text {if }0< \rho \le \rho _{\text {inf}}\\ c_0 + c_1 \rho + c_2 \rho ^2 + c_3 \rho ^3 & \text {if }\rho _{\text {inf}}< \rho \le \rho _{\text {sup}}\\ \alpha \rho & \text {if } \rho _{\text {sup}} < \rho \le 1 \end{array}\right. } \end{aligned}$$

The density thresholds $\rho _\textrm{inf}$ and $\rho _\textrm{sup}$ determine the local bone volume fraction ranges for trabecular, cortical and intermediate elements. The constants $c_0$, $c_1$, $c_2$ and $c_3$ were determined such that continuity and differentiability of the density function was ensured at $\rho _\textrm{inf}$ and $\rho _\textrm{sup}$ (Table 2).

**Table 2:** Parameters for density function according to Gerber et al. (2025)

$\boldsymbol{\rho _{\textrm{inf}}}$	$\boldsymbol{\rho _{\textrm{sup}}}$	$\boldsymbol{\alpha }$	$\textbf{k}$	$\mathbf {c_0}$	$\mathbf {c_1}$	$\mathbf {c_2}$	$\mathbf {c_3}$
0.45	0.85	2.203	1.91	6.967	-37.439	64.344	-32.177

## Appendix 3: Load case complexity

This section contains additional data visualising the impact of load case complexity on optimisation outcomes. Figure 8 presents the optimised forces and moments as well as corresponding inference results from statistical post-hoc analysis for optimisation approaches including varying model complexity. It should be noted that for F-XYZ all moments were set to zero by definition.

Similarly, for *F*–*Z* only an axial force was fitted. The influence of load case complexity on the objective function is depicted in Fig. 9.

Figure 10 shows the overlay of the B-splines presented in Figure 3b with the original data curves, confirming adequate representation of the key characteristics of the underlying dataset by the splines.

## Appendix 4 Local stress state analysis

For each element, the principal stresses in the optimised loading state for the standard model (STD) were identified and sorted in ascending order according to their absolute value. By analysing the signs of these principal stresses, eight distinct stress states arise in three-dimensional problems (+++, −++, − − +, +−+, ++−, −+−, − − −, +− − ). Figure 11 presents the local distribution of the stress states in the sagittal view of a typical distal tibia sample. A compressive maximal principal stress was observed in 99 % of all elements. In 63 %, all three stress eigenvalues had a negative sign. These regions were predominantly located in the sample’s core and at the proximal and distal boundary layers. Elements in the vicinity of the sample’s surface showed one or more tensile principal stress components due to the Poisson effect.

## Code availability

The code for FE model generation, inverse bone remodelling and data processing is shared open source on zenodo (https://doi.org/10.5281/zenodo.20280518).

## Materials availability

See data availability.
